# *QuickStats: *Death Rates* for Pedestrians Involved in Collision with Motor Vehicles,^†^ by Sex and Urbanization Level^§^ — National Vital Statistics System, United States, 2021

**DOI:** 10.15585/mmwr.mm7232a7

**Published:** 2023-08-11

**Figure:**
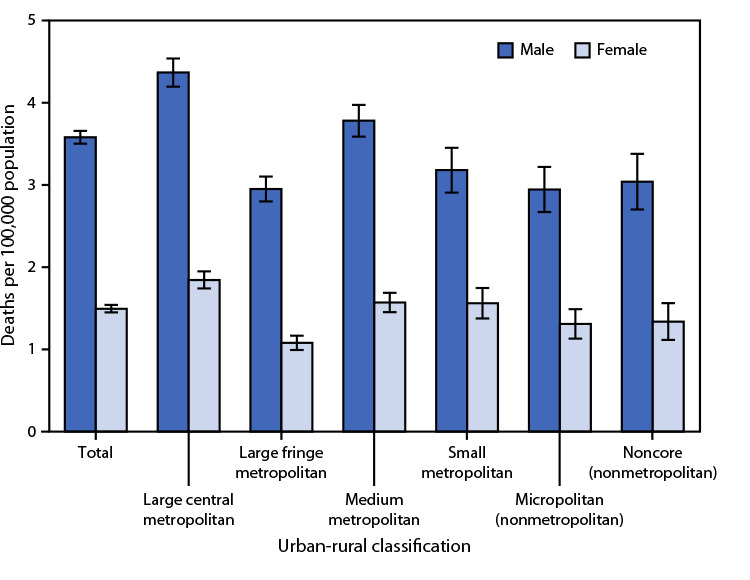
In 2021, death rates for pedestrians involved in collision with motor vehicles were 3.6 per 100,000 population for males and 1.5 for females. Rates were higher for males than for females at each urbanization level. Rates were the highest for males (4.4) and females (1.8) in large central metropolitan areas.

**Source:** National Vital Statistics System, Underlying Cause of Death, 2018–2021, https://wonder.cdc.gov/ucd-icd10-expanded.html

